# Adenovirus VA RNAI Blocks ASC Oligomerization and Inhibits NLRP3 Inflammasome Activation

**DOI:** 10.3389/fimmu.2019.02791

**Published:** 2019-11-28

**Authors:** Mahmoud Darweesh, Wael Kamel, Mikhail A. Gavrilin, Göran Akusjärvi, Catharina Svensson

**Affiliations:** ^1^Department of Medical Biochemistry and Microbiology, Uppsala University, Uppsala, Sweden; ^2^Department of Microbiology and Immunology, Al-Azhr University, Assiut, Egypt; ^3^Department of Internal Medicine, The Ohio State University, Columbus, OH, United States

**Keywords:** adenovirus, inflammasome, VA RNAI, PKR, proinflammatory cytokines, NLRP3, ASC

## Abstract

Virus infected immune cells can rapidly respond to the invader by activating the inflammasome and as a consequence release proinflammatory cytokines and eventually die by pyroptosis. In human adenovirus-5 (Ad5) infected THP-1 cells, inhibition of NLRP3 inflammasome activation was demonstrated by a decreased secretion of HMGB1 and matured forms of caspase-1and IL-1ß. An Ad5 mutant virus defective in expression of the non-coding VA RNAI failed to inhibit the NLRP3 inflammasome and in addition displayed formation of ASC specks and increased cell lysis. Importantly, *in vitro* synthesized VA RNAI was able to inhibit the NLRP3 inflammasome activity in THP-1 cells in the absence of an Ad5 infection, suggesting that VA RNAI binding to PKR and blocking its function is sufficient for inhibition of the NLRP3 inflammasome. Although the inhibition of NLRP3 inflammasome activation required the phylogenetically conserved base paired tetranucleotide sequence in the central stem of VA RNAI, we demonstrate that PKR binding to VA RNAI primarily protected the apical stem, but not the tetranucleotide sequence itself. VA RNAI did not influence the interaction between PKR and NLRP3. In contrast, we describe a novel interaction between PKR and ASC and further show that VA RNAI inhibited ASC phosphorylation and oligomerization. Collectively, our results indicate a novel role for Ad5 VA RNAI as an inhibitor of NLRP3 inflammasome activation by targeting the cellular pro-inflammatory protein PKR.

## Introduction

Human adenovirus-5 (Ad5) produces massive amounts of a specific class of small non-coding RNAs, the so-called virus-associated (VA) RNAI and VA RNAII [reviewed in (1)]. These multifunctional RNAs are required for efficient virus multiplication by targeting the host cells' innate immune response. One of the best characterized function of VA RNAI is as a competitive substrate for the interferon-inducible double-stranded RNA-dependent protein kinase (PKR). PKR, which is one of the key defense players in most virus infections (2), activates inflammatory cell signaling pathways and shuts down viral and cellular translation through phosphorylation of translation initiation factor eIF2α. To maintain an efficient translation during an adenovirus infection, the apical stem of VA RNAI binds to PKR, while the central domain inhibits PKR activation by preventing autophosphorylation (3–5). Interestingly, the recently described crystal structure of VA RNAI demonstrates that the apical stem and the well-conserved tetra nucleotide stem structure are both necessary and sufficient for PKR inhibition *in vitro* (6), possibly suggesting that the majority of the central region may fulfill additional functions *in vivo*. We and others have demonstrated that the terminal stem of the VA RNAs is processed into small viral microRNAs by the cellular Dicer enzyme, generating the so-called mivaRNAs (7) that target the RNAi/miRNA pathways in infected cells (8–12).

In addition to its effect on obstructing translation initiation through depletion of active eIF2α, PKR is also essential for mounting an antiviral response after a virus infection (13, 14). Activated PKR mediates the activation of mitogen-activated protein kinases (MAPKs) (14, 15), the inhibitor of κB (IκB) kinase (IKK) (16, 17) and IFN-β-promoter stimulator 1 (IPS-1) signaling (18). Activation of these pathways leads to enhanced expression of important transcription factors, like AP1 (19), NF-κB (17), and interferon regulatory factor 3 (IRF3) (18), which are involved in activation of genes encoding proinflammatory cytokines and IFNs.

A virus infection will trigger the assembly of cytosolic multiprotein inflammasome complexes, of which the NLRP3 (NOD-like receptor family, pyrin domain containing three) inflamamasome is the best characterized (20, 21). The NLRP3 sensor protein forms a multimeric complex with the ASC (apoptosis-associated speck-like protein containing a caspase recruitment domain) adaptor protein and recruits the effector molecule procaspase-1. Autocleavage leads to production of the active caspase-1 that in turn cleaves proinflammatory cytokines, such as pro-IL-1β and pro-IL-18 to generate active IL-1β and IL-18, which are subsequently secreted from the infected cell (22, 23). Active IL-1β is one of the key inflammatory cytokines in mounting an immune response and is implicated in many human diseases (24). Activation of the inflammasome also leads to extracellular release of the nuclear high mobility group box 1 (HMGB1) protein, which is a nuclear protein involved in regulation of transcription (25). The release of the proinflamatory HMGB1 protein has been shown to play a role in several inflammasome-related diseases and autoimmune disorders (25).

Interestingly, PKR has been suggested to directly enhance inflammasome activation and release of HMGB1. Peritoneal macrophages obtained from PKR-deficient mice released significantly lower amounts of HMGB1 after poly (I:C) treatment compared to macrophages from normal mice (26). PKR was also shown to interact with several inflammasome components (26). Moreover, cells deficient in the negative regulator of PKR, p58IPK showed increased inflammasome activation and cytokine secretion (27). However, opposite results have suggested that PKR is dispensable for inflammasome activation and secretion of active proinflammatory cytokines (28), and even that the PKR kinase activity had a repressive effect, rather than an enhancing effect, on inflammasome activity (28). Despite this discordance, numerous investigations have emphasized an important role of PKR in different inflammatory diseases (29, 30), although both suppressive and stimulatory activities have been described for malignant conditions (31). Thus, the outcome may be context dependent or rely on different mechanisms.

A virus infection expresses a multitude of signals that may trigger inflammasome activation by a variety of upstream receptors. An adenovirus infection is no exception and early steps of inflammasome activation occur when the virus penetrates the endosomal membrane (32), and cytosolic DNA sensors recognize the viral DNA through AIM2 (33, 34). Also the cGAS/STING pathway (35, 36) has been shown to recognize adenovirus DNA and trigger an antiviral response (37), although this does not appear to have an impact on viral replication efficiency.

Although inflammasome activation in adenovirus infected cells has been demonstrated experimentally (38), the overall efficient replication of adenovirus *in vivo* suggests that the virus has evolved factors suppressing inflammasome activation. For example, the Ad5 protein VII has the capacity to sequester HMGB1 to the nucleus thereby decreasing the extracellular secretion of the HMGB1 protein (39). However, an adenoviral factor interfering directly with inflammasome activation has until now not been described. Since the Ad5 VA RNAI is a well-characterized inhibitor of PKR activation we decided to test whether VA RNAI could function as a suppressor of inflammasome activation. In our experimental setup we aim to exclude early responses to incoming adenovirus DNA and focus on events during the late phase of infection when the expression of VA RNAI is at its maximum. By using this approach we could show that VA RNAI, indeed, blocked the activation of the NLRP3 inflammasome and thereby reduced the proteolytic activation of caspase-1 and also the extracellular release of HMGB1 and the active forms of caspase-1 and IL-1ß.

## Results

### Wild Type Ad5, but Not a Virus Lacking VA RNAI Expression Blocks HMGB1 Release in NLRP3 Inflammasome-Activated THP-1 Cells

**Figure 1:**
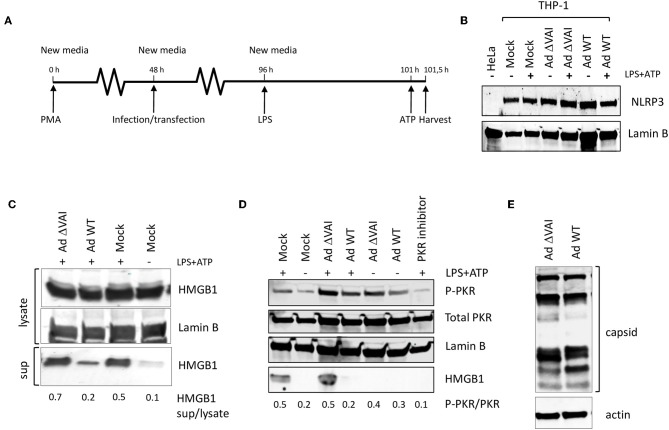
VA RNAI-dependent inhibition of HMGB1 release in Ad5-infectend THP-1 cells. **(A)** Outline of experimental setup to differentiate THP-1 cells prior to infection. Potential inflammatory cytokines released as a response to the early phase of the virus infection were removed by change to new media and the NLRP3 inflammasome assembly induced by the classical TLR4 agonist LPS, and ATP. **(B)** Western blot analysis of NLRP3 accumulation in THP-1 cells after the experimental setup in **(A)**. **(C)** Western blot analysis of inflammasome-activated (LPS + ATP treated) THP-1 cells infected with either with Ad WT or Ad ΔVAI showing a VA RNAI-dependent decrease of extracellular release of HMGB1. **(D)** Western blot analysis showing that the increased release of HMGB1 in inflammasome activated Ad ΔVAI-infected THP-1 cells correlates with increased phosphorylation of PKR. **(E)** Western blot analysis of Ad5 capsid proteins in Ad WT or Ad ΔVAI-infected THP-1 cells demonstrating phenotypically similar translation efficiency. In all panels actin was used as a loading control. Lysate; total cell extracts, sup; secreted proteins in the growth medium, PKR Inhibitor; PKR inhibitor 527450), Mock; uninfected cells treated similarly to infected cells. The data ratio given in **(C,D)** represent the mean ± SE from three independent experiments.

Since several reports have suggested that PKR activation may have a role in inflammasome regulation we tested whether the Ad5 VA RNAI, which is a well-characterized suppressor of PKR during an adenovirus infection, has an inhibitory effect on inflammasome activation. THP-1 cells were differentiated with PMA to obtain macrophage-like cells, and then infected with either wild type Ad5 (Ad WT), or the dl705 virus (40), which is an Ad5 mutant virus defective in VA RNAI expression (here referred to as Ad ΔVAI). At 48 h post-infection (hpi), growth media was replaced to remove potential inflammatory cytokines released as a response to the early phase of the virus infection (32), and NLRP3 inflammasome assembly and activation induced by the classical TLR4 agonist LPS, and ATP (Figure 1A). Expression of NLRP3 in THP-1 cells at the end-point of the experimental setup was relatively unaffected by inflammasome activation and adenovirus infection (Figure 1B). NLRP3 inflammasome activation was thereafter monitored by analyzing HMGB1, both in the cytosol and released to the growth media (Figure 1C). In uninfected cells (Mock), activation of the inflammasome by LPS and ATP resulted in the expected secretion of HMGB1, with only a minor increase in the intracellular levels of HMGB1 (Figure 1C). In Ad WT-infected cells, the amount of HMGB1 released to the media was significantly reduced as compared to activated mock infected cells (Figure 1C) suggesting a suppressive effect of the virus infection. A reduced HMGB1 release was not observed in the Ad ΔVAI-infected cells (Figure 1C) indicating that VA RNAI expression was required to prevent HMGB1 release in the infected cells. A similar suppression of HMGB1 release by Ad WT virus was observed in cells where the secondary signal ATP was replaced by nigericin (data not shown).

A single mechanism for activation of PKR has not yet been described (41) but is generally regarded to require a homodimerization and autophosphorylation. The involvement of PKR has been demonstrated during the early steps of inflammasome activation (26) and inhibition of PKR phosphorylation using PKR Inhibitor 527450 (42) decreased HMGB1 secretion in activated THP-1 cells (Figure 1D). The release of HMBG1 in inflammasome activated, AdΔVAI-infected cells correlated with a strong increase in the level of phosphorylated PKR. A slight increase in phosphorylated PKR was also seen in inflammasome activated, Ad WT-infected cells although strongly reduced HMBG1 release was observed. Overall, compared to unstimulated THP-1 cells, adenovirus infections caused increased phosphorylation of PKR, but the increased HMBG1 release was only seen after inflammasome activation and in the absence of VA RNAI (Figure 1D). This indicated that VA RNAI could interfere with inflammasome activation by regulating PKR activation in THP-1 cells.

In adenovirus infected human cell lines of epithelial and fibroblast origin VA RNAI has a critical role to rescue viral protein synthesis by preventing the translational inhibition caused by PKR-dependent phosphorylation of eIF2α [reviewed in (1, 43)]. To exclude that the VA RNAI-dependent reduction in HMGB1 release was due to a reduced translational efficiency in Ad ΔVAI-infected cells, late viral protein production was compared in Ad WT and Ad ΔVAI-infected THP-1 cells. As shown in Figure 1E, no significant difference in viral late protein accumulation was observed between Ad WT and Ad ΔVAI, suggesting that the translational machinery in Ad ΔVAI-infected THP-1 cells was essentially intact. The result further implies that the observed inhibition of HMGB1 release in Ad WT-infected cells is not related to a general VA RNAI-dependent effect on late viral protein expression, but rather due to specific function of VA RNAI.

### Active Inflammasome Formation and Secretion of Mature Proinflammatory Cytokines in Ad WT-Infected THP-1 Cells Is Inhibited by VA RNAI

**Figure 2:**
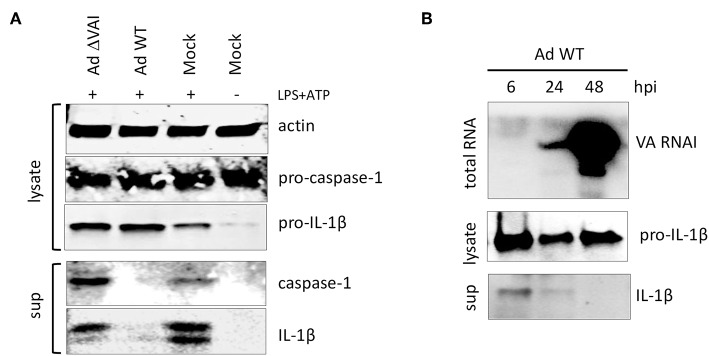
VA RNAI-dependent inhibition of mature caspase-1 and IL-1β secretion. **(A)** Western blot analysis of NLRP3 inflammasome-activated (LPS + ATP treated) THP-1 cells infected with either with Ad WT or Ad ΔVAI demonstrating a VA RNAI-dependent decrease of extracellular release of mature caspase-1 and Il-1ß. **(B)** Inhibition of release of Il-1ß (analyzed by Western blot) correlating to the increase in VA RNAI accumulation (analyzed by Northern blot) during the late stage of Ad WT infection of NLRP3 inflammasome activated THP-1 cells. The analysis was done at the indicated time points of infection (hpi). Lysate; total cell extracts, sup; secreted proteins in the growth medium, Mock: uninfected cells.

Activation of the inflammasome is, in addition to the release of HMBG1, also characterized by autocleavage of pro-caspase-1 into the active caspase-1 p20 subunit, which subsequently targets pro-IL-1ß to generate the mature IL-1ß (Figure 2A). Similar to the effect on HMGB1 release, an Ad WT infection inhibited the release of mature caspase-1 and IL-1ß subunits into the media (Figure 2A). In contrast, infection with Ad ΔVAI did not reduce the accumulation of secreted and proteolytically matured caspase-1 and IL-1ß (Figure 2A), corroborating the hypothesis that VA RNAI is required for the suppression of inflammasome activity.

VA RNAI reaches its maximal level of expression at the late stage of infection (44). Thus, it can be predicted that the inhibitory effect on cytokine secretion would reach its maxima when sufficient amounts of VA RNAI have accumulated. Northern blot analysis of the temporal accumulation of VA RNAI in activated THP-1 cells infected with Ad WT showed an inverse correlation of VA RNAI accumulation and IL-1ß secretion (Figure 2B). At 48 hpi, when VA RNAI levels were highest the level of secreted IL-1ß was undetectable (Figure 2B).

### Inhibition of Pyroptotic Cell Death and ASC Speck Formation in Ad WT-Infected Cells Are Dependent on VA RNAI

**Figure 3:**
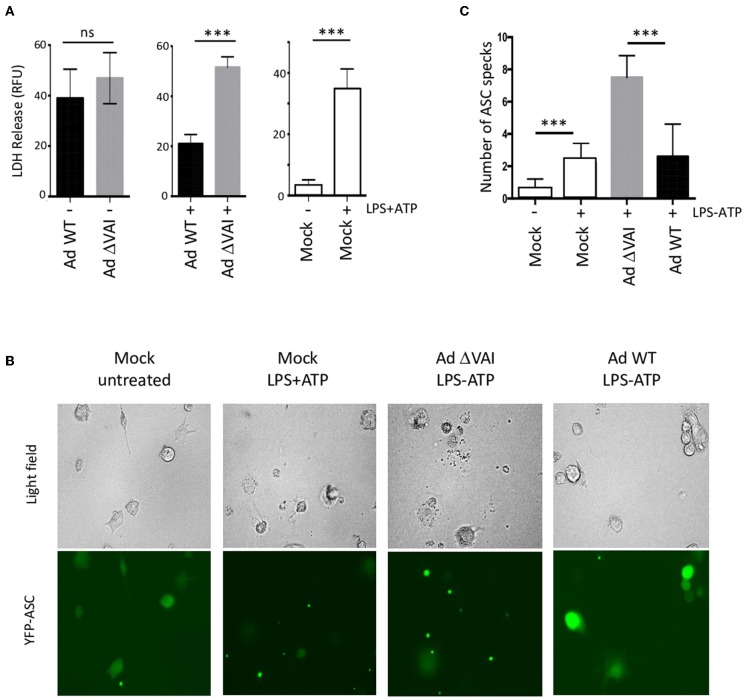
Reduced (pyroptotic) cell death in AdWT-infected THP-1 cells. **(A)** Quantification of LDH release in untreated or NLRP3 inflammasome-activated (LPS + ATP treated) THP-1 cells, either uninfected (right panel) or following infection with Ad WT or Ad ΔVAI demonstrating a VA RNAI-dependent suppression of cell death in the inflammasome activated THP-1 cells. LDH release from infected, but unstimulated cells was determined at 48 hpi (left panel). LDH release from infected cells following inflammasome activation was performed according to the experimental setup in Figure 1A (middle panel). LDH release was quantitated by a NAD^+^ reduction assay (relative fluorescent unit; RFU). **(B)** VA RNAI-dependent reduction of ASC speck formation in NLRP3 inflammasome-activated THP-1 cells expressing YFP-ASC, either uninfected (mock) or infected with Ad WT or Ad ΔVAI. For comparative reasons, images have been selected where the total number of cells/specs are similar. **(C)** Quantitation of the number of ASC specks seen in **(B)**. For each sample, numbers represent an average of specks counted from 10 individual fields. The quantitated data represent the mean ± SE from three independent experiments. Results are depicted as mean ± SEM. Statistical analysis was performed by Unpaired *t*-test ***p < 0.001; n.s., not significant.

A late consequence of caspase-1 and inflammasome activation can be cell death through pyroptosis (45). In dying cells, the release of lactate dehydrogenase (LDH) to the growth media due to loss of membrane integrity can be quantitated as a measure of cell death. To test whether the decreased secretion of HMGB1, IL-1ß, and caspase-1 in Ad WT-infected cells (Figures 1C,D, 2A) correlated with reduced cell death, we quantified the release of LDH in Ad WT or Ad ΔVAI-infected THP-1 cells. In uninfected THP-1 cells, inflammasome activation resulted in an ~10-fold increase in LDH release. At 48 hpi, but in the absence of inflammasome activation, both AdWT and AdΔVAI had released similar levels of LDH, probably through the overall cytopathic effect. To specifically address the effect of the inflammasome in infected cells, growth media was removed at 48 hpi and the inflammasome was activated by LPS and ATP (as described in Figure 1A). New release of LDH was then observed to be significantly less in AdWT infected cells compared to AdΔVAI-infected cells (Figure 3A), suggesting that VA RNAI protected cells from pyroptotic cell death.

Cell death by pyroptosis occurs after recruitment of the adaptor protein ASC (apoptosis-associated speck-like protein containing a carboxy-terminal caspase-recruitment domain) and the effector procaspase-1 to the sensor protein NLRP3. The activated inflammasomes activation will lead to formation of so called ASC specks, which will immediately be followed by cell death (46). However, extracellular ASC specks prevails sufficiently long to be counted as a measure of inflammasome activation. To directly analyze specks formation, we infected THP-1 cells stably expressing a YFP-tagged ASC (47) with Ad WT or Ad ΔVAI, and induced the NLRP3 inflammasome with LPS+ATP. The result is shown in Figure 3B and a quantification of ASC speck formation shown in Figure 3C. In uninfected and untreated THP-1 cells, very few specs were detected and the YFP-ASC protein was uniformly distributed throughout the cell (Figure 3B). A similar pattern was observed in Ad WT-infected cells. This was in stark contrast to Ad ΔVAI-infected cells, where YFP-ASC formed almost four-times the number of specks seen in activated mock cells or Ad WT infected cells (Figure 3C). Taken together these observations demonstrated that VA RNAI blocks formation of an active NLRP3 inflammasome.

### Ectopic Expression of Adenovirus VA RNAI Is Sufficient to Inhibit NLRP3 Inflammasome Activity

**Figure 4:**
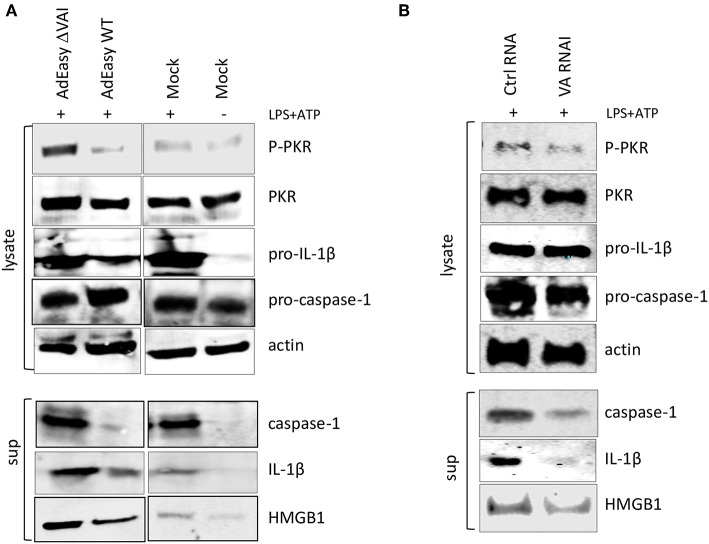
Ectopic expression of adenovirus VA RNAI is sufficient to inhibit the release of pro-inflammatory cytokines. **(A)** Western blot analysis of NLRP3 inflammasome-activated (LPS + ATP treated) THP-1 cells infected with replication deficient AdEasyWT or AdEasyΔVAI demonstrating a VA RNAI-dependent decrease of caspase-1, Il-1ß, and HMGB1 release. **(B)** Western blot analysis demonstrating the inhibition of caspase-1, Il-1ß, and HMGB1 release in NLRP3 inflammasome-activated (LPS+ATP treated) THP-1 cells transfected with *in vitro* transcribed VA RNAI. Ctrl RNA; *in vitro* transcribed RNA of the same size as VA RNAI, but with an unrelated sequence.

To determine whether VA RNAI was sufficient to inhibit inflammasome activation in the absence of other virus components we transduced activated THP-1 cells with the AdEasy vector expressing either the wild type VA RNAI gene (AdEasy WT) or a transcriptionally defective VA RNA I gene (AdEasy ΔVAI) (48). The non-replicating AdEasy virus vector system (49) allows for an efficient expression of VA RNAI in the absence of other viral gene products or viral DNA replication. Compared to AdEasy ΔVAI, AdEasy WT suppressed the release of HMGB1, caspase-1, and IL-1ß (Figure 4A). In agreement with our previous data, THP-1 cells transduced with AdEasy WT also reduced the phosphorylation of PKR (Figure 4A). Taken together, these results suggest that VA RNAI, in the absence of other viral gene products is sufficient to inhibit the activity of the NLRP3 inflammasome.

To further prove this point, PMA differentiated THP1 cells were transfected with *in vitro* transcribed VA RNAI or a control RNA of the same length, but of unrelated sequence. Twelve hours post-transfection cells were stimulated with LPS + ATP to activate the NLRP3 inflammasome. Although transfection rarely reaches the same high efficiency as virus transduction, the *in vitro* synthesized VA RNAI showed a clear inhibitory effect on HMGB1, caspase-1, and IL-1ß release compared to the *in vitro* synthesized control RNA (Figure 4B). Importantly, the *in vitro* synthesized VA RNAI also showed a reduction of PKR phosphorylation without affecting the total amount of PKR.

### VA RNAI Binding to PKR Is Essential for the Inhibition of NLRP3 Inflammasome Activity

**Figure 5:**
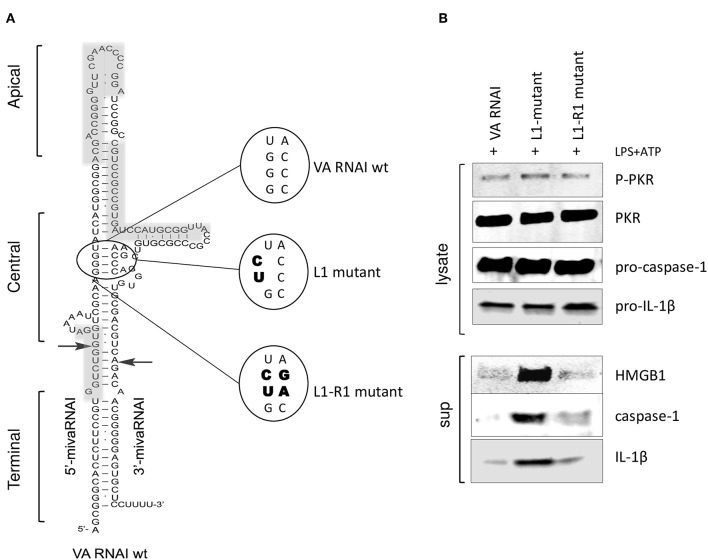
PKR-binding to VA RNAI is required for the inhibitory effect on pro-inflammatory cytokine release. **(A)** Schematic diagram showing the structure of Ad5 wild type VA RNAI, the location of the L1 mutant harboring a mutation disrupting the base-pairing in the conserved central domain essential for PKR binding and the L1-R1 mutant harboring a compensatory mutation restoring base-pairing in the central domain. Gray boxes represent the RNA sequences identified by HITS-CLIP analysis as protected by PKR binding (see also Supplementary Figure 1). **(B)** Western blot analysis demonstrating the inhibition of caspase-1, Il-1ß, and HMGB1 release in NLRP3 inflammasome activated (LPS + ATP treated) THP-1 cells transfected with *in vitro* transcribed VA RNAI, the inactive mutant L1 or the L1-R1 compensatory mutation.

The correlation between the secondary structure of adenovirus VA RNAI (Figure 5A) and its regulation of PKR activity has been thoroughly investigated (43). Base-pairing of the highly conserved central domain and the ssRNA and dsRNA character of the apical structure in VA RNAI (Figure 5A) are critical for the VA RNAI—PKR interaction and VA RNAI function *in vivo* and *in vitro* (3–5). Using high-throughput sequencing of RNA isolated by UV-crosslinking and PKR immunoprecipitation (HITS-CLIP) combined with bioinformatics analysis we identified the sequences protected by PKR in Ad5 VAI infected THP-1 cells (Figure 5A, gray boxes). The result demonstrates that the apical stem and loop appears to be the most prominent binding site for PKR (Supplementary Figure 1). Although VA RNAII-folds into a similar hairpin structure as VA RNAI, the PKR HITS-CLIP results for VA RNAII shows significantly less reads, which furthermore, only map to the terminal stem region of VA RNAII (Supplementary Figure 1). This result is in agreement with the observation that VA RNAII can only to a very limited extent secure viral translation during a lytic adenovirus infection.

A mutation disrupting base-pairing of a conserved pair of tetra-nucleotides, GGGU and ACCC (L1 mutant, Figure 5A) diminish the ability of VA RNAI to rescue PKR-induced inhibition of translation (3, 4). However, VA RNAI regains most of its activity when the base pairing is restored by introducing compensatory mutations (L1-R1, Figure 5A). To verify that the interaction of VA RNAI with PKR was also required for the observed inhibitory effect on the NLRP3 inflammasome, the Ad5 VA RNAI wild type, the VA RNAI L1 and VA RNA L1-R1 mutants (Figure 5A) were *in vitro* transcribed and transfected into THP-1 cells (Figure 5B). Both wild type VA RNAI and VA RNAI L1-R1 inhibited the release of HMGB1, caspase-1, and IL-1ß, whereas the VA RNAI L1 mutant with a disrupted base-pairing failed to do so (Figure 5B). Taken together, these observations support the hypothesis that VA RNAI exerts its inhibitory effect on the NLRP3 inflammasome through binding to PKR.

### VA RNAI Blocks ASC Oligomerization

**Figure 6:**
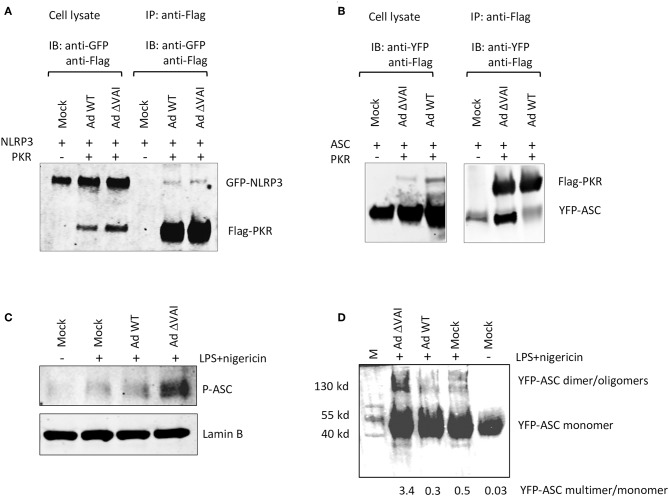
VA RNAI blocks ASC phosphorylation and oligomerization. HEK293 cells stably expressing YFP-ASC and co-transfected with plasmids expressing Flag-tagged PKR and a GFP-tagged NLRP3 fusion protein were infected with Ad WT or Ad ΔVAI and the cell lysates immunoprecipitated with anti-Flag antibodies. **(A)** Western blot analysis of coprecipitated NLRP3 and PKR using anti-GFP and anti-Flag-antibodies, respectively demonstrating a VA RNAI-independent interaction between NLRP3 and PKR. **(B)** Western blot analysis of coprecipitated ASC and PKR using anti-YFP and anti-Flag-antibodies, respectively, demonstrating a VA RNAI-dependent inhibition of the interaction between ASC and PKR. **(C)** Western blot analysis of phosphorylated ASC using a phospho-Tyr146-specific antibody demonstrating a VA RNAI-dependent inhibition of ASC phosphorylation. **(D)** Triton-X100 insoluble THP-1 cell pellets cross-linked with 4 mM disuccinimidyl suberate to preserve ASC oligomers. Samples dissolved in SDS sample buffer were subjected to Western blot analysis to detect ASC demonstrating a VA RNAI-dependent inhibition of ASC oliogmerization. The data ratio was calculated as the sum of oligomers and dimers vs. monomers.

PKR has previously been shown to interact with NLRP3, and also to be required for reconstitution of an active inflammasome complex in transfected HEK293 cells (26). Here we have shown that VA RNAI does not affect the total amount of PKR, but do, albeit to a varying degree, reduce the accumulation of the activated, phosphorylated form of PKR. Therefore, VA RNAI could theoretically block an essential interaction between PKR and component(s) of the inflammasome and/or inhibit the PKR-dependent phosphorylation of inflammasome target(s). To address the different possibilities, we analyzed the effect of VA RNAI on the interaction of inflammasome components in HEK293 cells stably expressing a YFP-ASC protein and co-transfected with plasmids expressing a Flag-tagged PKR protein and a GFP-tagged NLRP3 fusion protein. Extracts from Ad WT and Ad ΔVAI-infected cells were immunoprecipitated with an anti-Flag antibody and the Western blot probed with primary antibodies to detect PKR and co-precipitated NLRP3. As shown in Figure 6A, we observed a weak interaction between PKR and NLRP3 in infected cells. However, no difference between Ad WT or Ad ΔVA infection in the relative binding between PKR and NLRP3 was observed (Figure 6A) suggesting that VA RNAI did not suppress inflammasome complex formation at the level of a PKR-NLRP3 interaction. When using the same co-immunoprecipitation strategy, a novel interaction between PKR and YFP-ASC was observed in Ad ΔVAI-infected cells (Figure 6B). Interestingly, the interaction was clearly reduced in the Ad WT-infected cells (Figure 6B). Notably, even in the absence of Flag-PKR, small amounts of YFP-ASC was detected (Figure 6B) suggesting that the YFP-ASC transgene was expressed at high levels or that the protein has a general stickiness.

It has previously been demonstrated that ASC phosphorylation is required for formation of ASC oligomers (50–52). As shown in Figures 3B,C, Ad WT, but not Ad ΔVAI can block formation of ASC specs, a result that suggests that VA RNAI interferes with either ASC phosphorylation and/or ASC oligomerization. Comparison of the level of ASC phosphorylation in Ad WT and Ad ΔVAI-infected THP-1 cells demonstrated less ASC phosphorylation in Ad WT infected cells (Figure 6C). To verify that this also reduced ASC oligomerization, we used a combination of Triton X100 extraction and disuccinimidyl suberate (DSS) crosslinking to obtain ASC oligomers (53, 54) from Ad WT and Ad ΔVAI-infected and NLRP3 inflammasome-activated THP-1 cells. In agreement with the VA RNAI-dependent inhibition of ASC phosphorylation (Figure 6C), and inhibition of specs formation (Figures 3B,C), we observed that Ad WT infection also reduced ASC oligomer formation (Figure 6D). Taken together, our results suggest that VA RNAI blocks the interaction between PKR and ASC to inhibit ASC phosphorylation and consequently ASC oligomerization.

### A VA RNAI Terminal Stem Chimera Expressing miR-146 Reduces TRAF6 Accumulation While Still Retaining the Ability to Inhibit the NLRP3 Inflammasome

**Figure 7:**
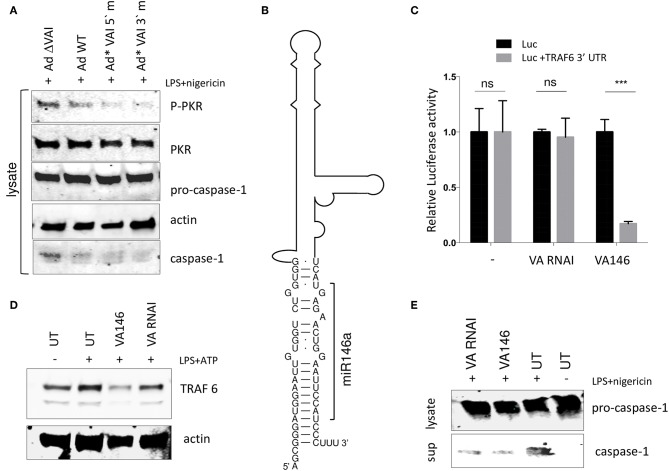
A VA RNAI terminal stem chimera expressing miR-146 reduces TRAF6 accumulation retains the ability to inhibit the NLRP3 inflammasome. **(A)** Western Blot showing that replication competent Ad5 viruses with mutations in either the VA RNAI 5′or 3′seed sequences (Ad*VAI 5′m and Ad*VAI 3′m) were as efficient as Ad WT in preventing phosphorylation of PKR and blocking the release of caspase-1. **(B)** Schematic diagram showing the sequence of the Ad5 VA RNAI/miR-146 chimera. **(C)** HEK293 cells co-transfected with *in vitro* transcribed wild type VA RNAI or VA146 and luciferase reporters containing or lacking the miR-146 binding site from the TRAF6 mRNA demonstrates a miR146 dependent-repression of luciferase activity. **(D)** Western blot analysis showing that transfection of *in vitro* transcribed VA146 down-regulates endogenous TRAF-6 protein expression in NLRP3 inflammasome activated (LPS + ATP treated) THP-1 cells. **(E)** Western blot analysis showing that *in vitro* transcribed VAI146 and wild type VA RNAI inhibit IL-1ß release in NLRP3 inflammasome activated (LPS + ATP treated) THP-1 cell to the same degree. The quantitated data represent the mean ± SE from three independent experiments. Results are depicted as mean ± SEM. Statistical analysis was performed by Unpaired *t*-test ***p < 0.001; n.s. not significant.

The results shown in Figure 5 are consistent with a model where the interaction between the apical stem and the central domain of VA RNAI, and PKR is responsible for the observed inhibition of the NLRP3 inflammasome. We and others have shown that the terminal stem of VA RNAI (Figure 5A) is processed into small viral microRNAs (the so-called mivaRNAs) with a capacity to interfere with the RNAi/miRNA pathways in infected cells. To test whether the mivaRNAs are involved in inflammasome activation, VA RNAI with mutations in the seed sequences (48) were analyzed for their ability to inhibit the inflammasome. As shown in Figure 7A, replication competent Ad5 viruses with mutations in either the 3′or 5′seed sequences (Ad^*^VAI 5′m and Ad^*^VAI 3′m) were at least as efficient as Ad WT in preventing phosphorylation of PKR and blocking the release of caspase-1 in THP-1 cells. Therefore, we concluded that the function of VA RNAI as an inhibitor of NLRP3 inflammasome activation is not dependent on the capacity of VA RNAI to produce native mivaRNAs.

This finding opened up the possibility to create a retargeted VA RNAI gene with the capacity to modulate a specific cellular gene through the RNAi/miRNA pathways. We selected the cellular miR-146, which has been shown to control TLR and cytokine signaling through a negative feedback regulatory loop involving down-regulation of IL-1 receptor-associated kinase 1 (IRAK) and TNF receptor-associated factor 6 (TRAF6) protein levels (55). The terminal sequence in Ad5 VA RNAI was replaced with the sequence of miR-146a (56) to produce a VA RNAI miR-146 chimera (VAI146; Figure 7B). After *in vitro* transcription, VAI146 was co-transfected with luciferase reporter constructs into HEK293 cells. As shown in Figure 7C, VA146 appeared to produce a functional miR-146 since a specific repression of luciferase activity was observed, but only when the miR-146 target sequence from the TRAF6 3′-UTR was inserted into the 3′ UTR of the luciferase reporter construct. Moreover, comparison of *in vitro* transcribed VA RNAI and VA146 after transfection of THP-1 cells showed that the endogenous TRAF6 protein levels were specifically reduced in VA146 transfected cells (Figure 7D). Importantly, the VA146 was as efficient as the wild type VA RNAI to inhibit the release of caspase-1, suggesting that a retargeted mivaRNA does not interfere with the ability of VA RNAI to inhibit inflammasome activation (Figure 7E).

## Discussion

Infection with human adenovirus results in activation of several innate immune activities in the infected cell. For example, activation of transcription factors, such as IRF3 and NF-κB can lead to transcription of IFN-ß and proinflammatory cytokines, dsRNA activation of PKR can ultimately shutdown translation and viral DNA can activate caspase-1 through the inflammasome signaling pathway. However, the virus triggers a number of countermeasures that are essential for the virus to establish a productive infection. For example, the E1A proteins block activation of interferon transcription (57, 58), VA RNAI prevents PKR activation (59, 60) and the viral histon-like protein VII binds to and sequesters HMGB1 to chromatin (39), thereby preventing its release and stimulatory effect on proinflammatory processes.

In previous work, adenovirus was shown to activate the NLRP3 inflammasome at early time points of infection through TLR9 sensing the virus double-stranded DNA after endosomal membrane penetration (32). In this work, we focused on how Ad5 copes with the proinflammatory response at late times of infection. To achieve this, the proinflammatory cytokines released to the growth media during the early phase of infection were removed. Instead, inflammasome activation was induced during the late stage of infection using the classical priming TLR4 agonist LPS and the inflammasome activator ATP.

Conflicting results have been presented regarding if and how PKR is involved in inflammasome activation. These results spans from no effect at all (61), to activation (26, 27, 62, 63) or inhibition (28) of inflammasome activity. Since Ad5 VA RNAI is a known inhibitor of PKR we set out to study the effect of VA RNAI on inflammasome activation. Our results strongly support the view that PKR has a role in NLRP3 inflammasome activation. We also describe the novel capacity of VA RNAI *per se* to inhibit PKR-dependent activation of the NLRP3 inflammasome. The data relies on three different approaches: Introduction of VA RNAI into THP-1 cells via; (i) infection with a replication competent adenovirus with or without the capacity to express VA RNAI; (ii) transduction of the VA RNAI gene using the replication defective AdEasy vector system, and (iii), transfection of *in vitro* transcribed wild type or mutant VA RNAI molecules. The first two approaches both demonstrated that VA RNAI was required to block LPS + ATP stimulated NLRP3 inflammasome activation. During the early phase of an adenovirus infection, viral DNA is recognized by AIM2, which recruits ASC to form an inflammasome complex (33). Thus, it is possible that VA RNAI also has an inhibitory effect on the AIM2 inflammasome, even if the cytokines secreted due to sensing of viral DNA were removed prior to analysis of inhibition in our study. Moreover, it is also possible that VA RNAI expressed by the AdEasy vector may dampen additional antiviral activities induced by the incoming viral DNA, possibly explaining the observed variation in pro-IL-1ß (Figure 4A). However, since VA RNAI is primarily expressed at the later time points of a lytic infection (44) (Figure 2B) its role as a potential AIM2 inflammasome regulator has not been addressed here. Importantly, our third approach, which involves introduction of *in vitro* synthesized VA RNAI, demonstrated that VA RNAI alone was sufficient to block the NLRP3 inflammasome as measured by secretion of HMGB1, and proteolytically processed caspase-1 and IL-1ß. VA RNAI was, in this approach produced using T7 RNA polymerase, a procedure that may cause unwanted transcription from the opposite strand of the template, resulting in production of dsRNA with potentially immune-stimulatory activities (64). PKR activation by dsRNA produced by T7 RNA polymerase transcription of a snoRNA gene was in fact recently demonstrated (65). However, we did not observe effect on the NLRP3 inflammasome either by a T7 RNA polymerase produced control RNA or a mutant VA RNA I gene previously described to lack PKR regulatory activity. Thus, we conclude that the potential production of a dsRNA from the templates used in this study did not mask the specific blocking activity of VA RNAI.

Notably, whereas AdWT infection blocked NLRP3 inflammasome activity compared to uninfected cells (Figure 1C), infection with a replication defective AdEasy vector appeared to potentiate the release of IL-1ß and HMGB1 compared to uninfected cells (Figure 4A). Therefore, it is possible that infection of THP-1 cells with a replication competent Ad5 may lead to expression of other viral factors that can interfere with proinflammatory signaling. One such example is the Ad5 protein VII, which has been shown to sequester HMGB1 in the nucleus (39).

The well-supported mechanism by which VA RNAI prevents PKR-induced translation inhibition describes a process where VA RNAI binds a monomer of PKR thereby preventing dimerization, autophosphorylation and thus activation [reviewed in (1, 43)]. The apical stem of VA RNAI is essential for the high affinity binding and inhibition of PKR activity, whereas the contribution of the central domain, including a formation of a pseudoknot is less clear (3–5, 66). The conserved tetranucleotide pair in VA RNAI, is on the other hand critical for PKR binding and inhibition of PKR activation both *in vivo* and *in vitro* (3–5). In the recently reported crystal structure of VA RNAI, the tetranucleotide pair together with the apical duplex stem was shown to be both necessary and sufficient to prevent PKR activation (6). Using a HITS-CLIP analysis of the VA RNAI—PKR interaction, we showed that sequences in the apical and central regions of VA RNAI was protected by PKR binding. However, no direct binding of PKR to the tetranucleotide base pairs was observed. Still, mutations in the tetranucleotide pair known to interfere with PKR inhibition also prevented the ability of VA RNAI to inhibit inflammasome activation (Figure 5). Moreover, a compensatory mutation that is known to restore inhibition of PKR also reverted the defect on the blocking of the inflammasome. The compensatory L1-R1 mutant (Figure 5A) was less effective in blocking caspase-1 and cytokine release compared to the wild type VA RNAI (Figure 5B), in agreement with its about half activity on inhibition of PKR activation (4). Since reduced phosphorylation of PKR was observed in parallel with VA RNAI-dependent inflammasome inhibition our data indicates that VA RNAI inhibition of NLRP3 inflammasome activation may function through the known inhibitory mechanism of PKR activation. The mechanism by which PKR activates inflammasome formation is under debate. Lu et al. (26) have demonstrated an interaction between PKR an NLRP3 and that PKR lacking kinase activity is compromised in inflammasome activation. In this report, we demonstrate that PKR makes a physical interaction also with ASC. We show that VA RNAI inhibited the interaction between PKR and ASC, but not the interaction between PKR and NLRP3 (Figure 6). Furthermore, the characteristic formation of specks caused by inflammasome stimulation was significantly reduced in the presence of VA RNAI (Figure 3). The formation of specks is a hallmark of active NLRP3 inflammasomes and requires phosphorylation of ASC and subsequent oligomerization of ASC (50). It was therefore an important observation that phosphorylation of ASC was blocked by a VA RNAI-dependent mechanism. A number of tyrosin kinases have been implicated in phosphorylation of ASC (51) and mutation of the highly conserved Tyr 146 appears to abrogate both ASC speck formation and inflammasome activation (50). PKR is characterized as a serine/threonine kinase, but the activation of the PKR kinase domain requires an intermolecular autophosphorylation of tyrosine residues when latent monomers of PKR dimerize (11). Thus, PKR is a dual specificity kinase, but so far the tyrosin phosphorylation has not been implicated in more than regulation of PKR dimerization (67).

It should also be noted that since other proteases are also able to cleave IL-1ß, some even independently of ASC, we cannot exclude that other mechanism with alternative dependence of PKR may be involved.

Collectively, our data supports a mechanism where PKR stimulates inflammasome activation and further that VA RNAI inhibits inflammasome activation by blocking an interaction between PKR and ASC, which would otherwise lead to ASC phosphorylation and oligomerization. As a consequence of inflammasome inhibition, VA RNAI limits the formation of the speck-like structures (pyroptosomes) containing ASC (Figure 3) thereby protecting cells from pyropthotic cell death. Although merely indicative in this report, it will be interesting to further explore the possibility to potentiate the inflammasome inhibitory activity of VA RNAI by constructing VA RNA-miRNA hybrids, such as the VA RNAI-miR-146 chimera (Figure 7), targeting other crucial components involved in inflammasome activation.

Aberrant activation of the inflammasomes has been implicated in different neurological, autoimmune, cardiovascular, and malignant diseases (68). In virus infections, proinflammatory responses may, during persistent infections lead to long term inflammation, thereby creating a risk for different health conditions. The result presented here that a small viral RNA molecule may dampen the inflammasome activity could potentially increase our understanding of how to curb inflammation.

## Materials and Methods

### Viruses and Plasmids

Viruses used in this study were wild type Ad5 (referred to as Ad WT), the VA RNAI^−^/VA RNAII^+^ mutant virus dl705 (40) (here referred to as Ad ΔVAI), and the replication defective viruses AdEasy WT and AdEasy ΔVAI (48). To create replication competent AdEasy viruses expressing the VA RNAI 5′ and 3′seed sequence mutations (48) we used homologous recombination to reintroduce the seed mutated VA RNAI genes into their normal chromosomal position at map units 30, thereby generating Ad^*^-VAI 5′m and Ad^*^VAI 3′m. Details about the cloning strategies are available on request. Plasmids expressing VA RNAI from a T7 promoter have previously been described (8). The Ad5 VA RNAI L1 and VA RNAI L1-R1-mutants with central stem domain mutations (Figure 5A) were chemically synthesized (Genscript) and cloned by the company into pUC19 as a XbaI/EcoRI restriction fragment. FLAG-tagged PKR (N-DDK (Flag®) were obtained from Sino Biological and GFP-tagged NLRP3 proteins (PEGFP-C2 NLRP3) from Addgene. A 940 base-pair fragment of the TRAF6 3′UTR, containing three binding sites for miR-146a, was cloned into the pMirGlo plasmid (Promega) as a PmeI-SacI restriction enzyme fragment. Briefly, cytoplasmic RNA from HeLa cells was converted into cDNA using the SuperScript III First-Strand Synthesis kit (Thermo Fisher Scientific company) and the TRAF6 3′UTR PCR was subsequently amplified using forward primer AAAGTGAAAATCACTACCGCCT and reverse primer GCGAGCTCGTACCAGACAACTTTAAATGGTGGA. The Ad5 VA RNAI L1 and Ad5 VA RNAI L1-R1-mutants, with central stem domain mutations (Figure 5A), and the Ad5 VA146 chimera (Figure 7A) were chemically synthesized (Genscript) and cloned by the company into pUC19 as an XbaI/EcoRI restriction fragment.

### Cell Lines

THP-1 cells (ATCC® TIB-202) were maintained as suspension cultures in RPMI 1640 media, supplemented with 10% fetal bovine serum (FBS), 100 IU/ml penicillin, 1 mg/ml streptomycin, 0.25 μg/ml amphotericin B, 1% ml of 100× non-essential amino acids (Gibco Thermo Fisher), 10 mM HEPES buffer, 1 mM sodium pyruvate, and 2 mM glutamine. Differentiation of THP-1 cells into monolayers of macrophage-like cells was achieved by seeding the cells on plates and stimulating for 24 h with 100 nM phorbol-12-myristate-13-acetate (PMA; EMD Chemicals). THP-1 cells stably expressing an shRNA directed against ASC (47) or stably expressing a YFP-ASC fusion protein were maintained in RPMI 1640 media, supplemented as above. HEK293 cells (ATCC) and HEK293 stably expressing TLR4-MyD 88 signaling along with YFP-ASC (69) (Gavrilin et al., personal communication) were maintained in DMEM containing 10% FBS, 100 IU/ml penicillin, 1 mg/ml streptomycin, and 0.25 μg/ml amphotericin B.

### Infections and NLRP3 Inflammasome Activation

At 48 h post-PMA treatment, monolayers of activated THP-1 cells were infected with 20 FFU/cell of the indicated adenoviruses. 48 hpi, the growth medium was replaced with serum free DMEM and the NLRP3 inflammasome activated by priming with 500 ng/ml of LPS (Invivogen) for 5 h followed by addition of either 5 mM ATP (Sigma-Aldrich) or 20 nM nigericin (Tocris) for 30 min. When indicated, the PKR Inhibitor 527450 (CAS 608512-97-6 – Calbiochem; Sigma-Aldrich), dissolved in DMSO, was added to THP-1 cells at a concentration of 0.1 μM for 2 h before activation of the NLRP3 inflammasome.

### Lysate and Supernatant Protein Collection

To analyse secreted proteins, the growth medium was collected and centrifugated at 14,000 × g for 15 min at 4°C to remove cellular debris. One ml of the supernatant was transferred to a fresh 1.5 ml tube containing 10 μl of StrataClean resin (Agilent Technologies). After 1 h on a rotating wheel at 4°C, proteins bound to the resin was collected by a brief centrifugation and thereafter eluted by resuspension in 30 μl 1 × Laemmli buffer. Total cell lysates were prepared by lysis of cells by incubation with 100 μl RIPA buffer for 1 h followed by transfer of the lysate to a 1.5 ml tube. Equal volume of 2X Laemmli buffer was added and the samples heated at 95°C for 5 min before separated on a 10% SDS-PAGE.

### Immunoblotting

Twelve microliters of supernatant or cell lysate were separated on a 4–12% Mini-Protean TGX gel (Bio-Rad) at 100 V for 1 h. Proteins were transferred onto a 0.2 μm nitrocellulose membranes (Li-Cor Biotechnology) at 100 V for 1 h. The membranes were blocked in 5% non-fat dry milk in PBS containing 0.2% Tween-20 for 1 h followed by incubation in PBS containing 5% BSA, 0.2% Tween-20, and either 1:1,000 rabbit polyclonal anti-Caspase-1 p20 (47), anti-IL-1β (70), anti-HMGB1 (abcam; ab18256), anti-PKR (phospho T446) (abcam; ab32036), anti-PKR (abcam; ab226819), anti-beta Actin antibody (abcam; ab8229), Anti-GFP antibody (abcam; ab6556), or 1:500 anti-Asc (Tyr-144) phospho-specific antibody (ECM Bioscience; AP5631) with rotation overnight at 4°C. The following day, donkey anti-rabbit IRDye 800CW and donkey anti-goat IRDye 680RD secondary antibodies (Li-Cor Biotechnology) were applied at a dilution of 1:15,000 with rocking for 1 h at room temperature. Membranes were imaged on a Li-Cor Odyssey CLx machine with auto exposure and high-quality setting.

### Lactate Dehydrogenase (LDH) Cytotoxicity Assay

A NAD^+^ reduction assay (Roche Applied Science) was used to quantitate LDH release from cells. The growth medium was collected and clarified by centrifugation at 400 g for 5 min and the LDH concentration in the medium was measured at 490 nm. Cell death was calculated by the formula: cytotoxicity (%) = [(sample-blank)/(positive control-blank) × 100], where blank indicates OD value form RPMI-1640 and positive control OD values determined as total LDH content in untreated THP1 cells lysed with 1% Triton X-100.

### ASC Speck Visualization Using YFP-ASC THP1 Cells

For microscopy, 10^6^ THP-1 cells stably expressing YFP-ASC were plated in a 6-well culture plate (Costar) in a total volume of 2 ml/well. Cells were infected as described above, and the NLRP3 inflammasome activated by LPS + ATP treatment. ASC specks were visualized using the EVOS M7000 Imaging System, inverted microscope using 480/20 nm excitation and detection at 520 nm emission under a 40X objective. Fluorescent images of ASC specks were quantified using Image J software (NIH, http://rsbweb.nih.gov/ij).

### High-Throughput Sequencing of RNA Isolated by UV-Crosslinking and Immunoprecipitation (HITS-CLIP) and Bioinformatic Analysis

HITS-CLIP and library preparation were performed essentially as described (71) with the following modifications: At 24 hpi, THP1 cells (mock or Ad WT) were washed twice in PBS and irradiated twice with 400 mJ/cm^2^ at 254 nm. Cell lysate were incubated with either anti-PKR (abcam) or the negative control antiGFP (abcam). Raw sequence reads were trimmed using Cutadapt (72) and mapped to the human Adenovirus 5 reference genome (AC_000008.1), obtained from NCBI using STAR (73) with option -outFilterMismatchNoverLmax 0.3 (to account for RNA damage caused by high dose of UV). Subsequent analysis was done using only uniquely mapped reads. Filtering out regions covered by both PKR and GFP HITS-CLIP libraries was done using BEDTools with intersect option. Specific PKR HITS-CLIP reads was visualized on Integrative Genomics Viewer (IGV) (74).

### Cross-Linking of ASC Dimers or Oligomers

ASC oligomers were purified as reported before (75). Briefly 15 × 10^6^ THP-1 cells stably expressing YFP-ASC were infected with indicated adenovirus. At 48 hpi, NLRP3 was activated as described, and the cells were then incubated on ice for 30 min in Buffer A (20 mM HEPES-KOH, pH7.5, 10 mM KCl, 1.5 mM MgCl2, 1 mM EDTA, 1 mM EGTA, one tablet of each/10 ml lysis buffer of complete™ EDTA-free Protease Inhibitor Cocktail (Sigma-Aldrich) and PhosSTOP phosphatase inhibitor (Sigma-Aldrich). Lysis was completed by shearing 30 times through a 21-gauge needle, and the lysates were cleared by centrifugation for 8 min at 1,800 × *g*, 4°C. The supernatants were diluted in a 1:1 ratio with buffer A and cleared once more by centrifugation at 2,000 × *g* for 5 min at 4°C. The supernatants were diluted with 1 volume of CHAPS buffer and the ASC oligomers pelleted by centrifugation at 5,000 × *g* for 8 min. Pellets were resuspended in 50 μl of CHAPS buffer containing 4 mM of disuccinimidyl suberate (dissolved in DMSO) and incubated for 30 min at RT to cross-link proteins. Following a final centrifugation at 5,000 × *g* for 8 min at 4°C, the pellets were resuspended in 20 μl of 2 × protein loading buffer, boiled for 5 min at 100°C and loaded onto Precast Protein Gels (Any kD™ Mini-PROTEAN® TGX™, CA #4569036). ASC was detected by Western blot using Anti GFP-specific antibody (Abcam – ab290) as described above.

### *In vitro* Transcription of WT and Mutant VA RNAI Genes

The template for the *in vitro* transcription of the VA RNA genes were PCR products produced using a forward primer (containing the T7 promoter sequence shown in bold) **ATAATACGACTCACTATAG**GGATGGAATTGTGGTCTGGTG and a reverse primer AAAAGGGATGGAATTCAGTTCT). *In vitro* transcription was done using the TranscriptAid T7 High Yield transcription kit (ThermoFisher) according to the manufacture's instructions. The RNA was purified by one round of phenol extraction and ethanol precipitation. The RNA was dissolved in sterile H_2_O and stored at −80°C.

### Luciferase Assay

Luciferase assay was performed with the Nano-Glo® Luciferase Assay System Reporter Assay System (Promega) according to the manufacturer's instructions. Both firefly luciferase and Nano luciferase activity were measured on Infinite M200 luminometer (Tecan) and the firefly activity was normalized to the Nano activity and presented as means from at least three biological replicates. Statistical analysis was performed on Prism6 (GraphPad Software, USA) using a two-tailed unpaired *t*-test. The value of *p* < 0.05 was considered statistically significant.

### Coimmunopreciptation

HEK293 cells stably expressing YFP-ASC and with intact TLR4/MyD88 signaling were seeded at 5 × 10^5^ cells/well in six-well plates. Cells were transfected with plasmids expressing Flag-tagged PKR (PKR cDNA ORF clone, human, N-DDK (Flag) tag, BioSite CA HG10080-NF) and GFP-tagged NLRP3 (pEGFP-C2-NLRP3, addgene CA 73955). Cells were lysed in lysis buffer (20 mM Tris HCl pH 8, 137 mM NaCl, 1% Nonidet P-40 (NP-40), 2 mM EDTA). Ten microliters of anti-FLAG-M2-conjugated-agarose (Sigma) was pre-incubated with 5% BSA in PBS to minimize non-specific interaction before added to 100 μl of the cell lysate in 500 μl of IB solution [5 mM Tris-Cl, 10 mM HEPES, pH 7.5, 10% glycerol, 50 mM KCl, 0.05% Triton X-100, 1 mM EDTA, 1 mM dithiothreitol, 1× complete protease inhibitor (Roche Diagnostics)]. Incubation was at 4°C with gentle rotation overnight after which the beads were collected, washed three times with 500 μl of IB solution for 10 min each. The beads were resuspended in 30 μl of 2X SDS sample buffer, boiled after which 20 μl of the elutates were subjected to Western blot using antibodies described above.

### Statistical Analysis

The data were analyzed using Prism Version 5.01 (GraphPad Software, La Jolla, CA). Parametric tests were used to compare different treatments with Unpaired *t*-test. The data are expressed as the mean ± standard error, and the differences were considered significant as indicated as follows: ^*^p < 0.05; ^**^p < 0.01; ^***^p < 0.001; ^****^p < 0.001; ns: not significant.

## Data Availability Statement

All datasets generated for this study are included in the article/Supplementary Material.

## Author Contributions

MD, GA, and CS designed the experiments, interpreted data, created models, and wrote the paper. MG provided material and critically reviewed the paper. MD and WK performed the experiments and provided materials.

### Conflict of Interest

The authors declare that the research was conducted in the absence of any commercial or financial relationships that could be construed as a potential conflict of interest.

## Supplementary Material

Supplementary Figure 1 HITS-CLIP analysis identification of the regions in VA RNA protected by the interaction with PKR. **(A)** Number of RNA sequence reads in VA RNAI and VA RNAII specifically co-precipitated by anti-PKR and aligned to the Ad5 reference genome (AC_000008.1; NCBI). **(B)** Genomic location of the VA RNA genes. **(C)** Location of the identified reads in the secondary structure of the VA RNA genes are shown in red. Nucleotides in bold in VA RNAI denotes the predominant reads in the apical region. Nucleotides in bold black in both VA RNAs shows the location of the tetranucleotide base pairs.
