# Supplementary figures and images for: Adenovirus VA RNAI Blocks ASC Oligomerization and Inhibits NLRP3 Inflammasome Activation
Source: Front Immunol. 2019 Nov 28;10:2791. doi: 10.3389/fimmu.2019.02791 (PMC6901988; doi:10.3389/fimmu.2019.02791)

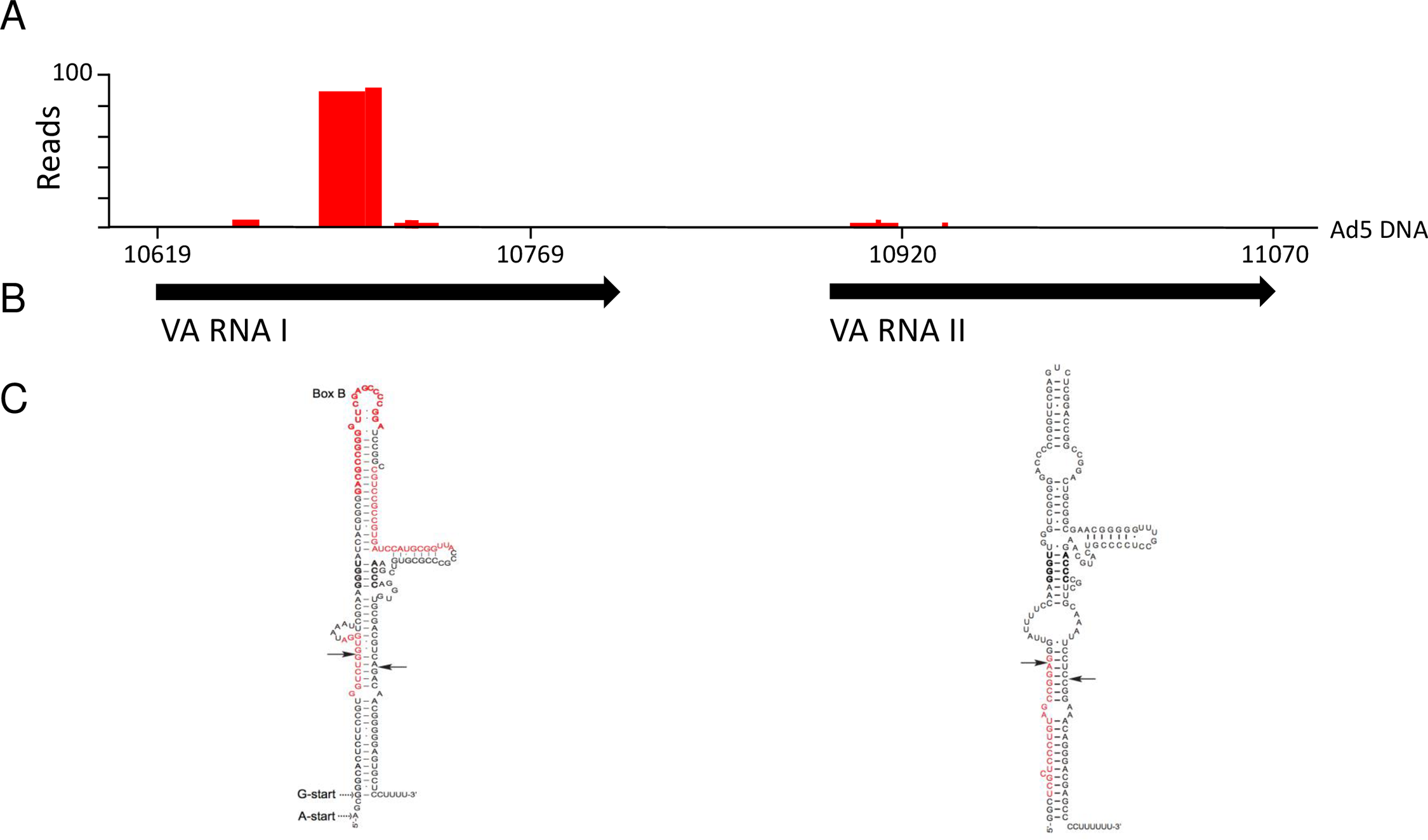

Supplement: Supplementary Figure 1 — HITS-CLIP analysis identification of the regions in VA RNA protected by the interaction with PKR. (A) Number of RNA sequence reads in VA RNAI and VA RNAII specifically co-precipitated by anti-PKR and aligned to the Ad5 reference genome (AC_000008.1; NCBI). (B) Genomic location of the VA RNA genes. (C) Location of the identified reads in the secondary structure of the VA RNA genes are shown in red. Nucleotides in bold in VA RNAI denotes the predominant reads in the apical region. Nucleotides in bold black in both VA RNAs shows the location of the tetranucleotide base pairs. [file Image_1.TIF]
